# The prevalence of *Borrelia miyamotoi* infection, and co-infections with other *Borrelia* spp. in *Ixodes scapularis* ticks collected in Canada

**DOI:** 10.1186/1756-3305-7-183

**Published:** 2014-04-15

**Authors:** Antonia Dibernardo, Tyler Cote, Nicholas H Ogden, L Robbin Lindsay

**Affiliations:** 1 Public Health Agency of Canada, Zoonotic Diseases and Special Pathogens, National Microbiology Laboratory, 1015 Arlington Street, Winnipeg, Manitoba, Canada; 2 Public Health Agency of Canada, Zoonoses Division, Centre for Food-borne Environmental and Zoonotic Infectious Diseases, Saint-Hyacinthe, Quebec, Canada

**Keywords:** *Borrelia miyamotoi*, Lyme disease, Real-time PCR, Co-infection

## Abstract

**Background:**

Blacklegged ticks, *Ixodes scapularis* are vectors of the tick-borne pathogens *Borrelia burgdorferi*, *Anaplasma phagocytophilum* and *Babesia microti.* Recently, the *I. scapularis*-borne bacterium *Borrelia miyamotoi* has been linked to human illness in North America. The range of this tick is expanding in Canada which may increase the potential for human exposure to these agents.

**Methods:**

In this study, 4938 *I. scapularis* ticks collected in 2012 were tested following a newly developed PCR-based testing protocol to determine the prevalence of infection with *B. miyamotoi* and other pathogens in *I. scapularis* in Canada.

**Results:**

*Borrelia miyamotoi* was detected in blacklegged ticks from all provinces except Newfoundland, although the infection prevalence was low (<1%). There was significant variation among provinces in the prevalence of infection of ticks with *B. burgdorferi* and *A. phagocytophilum,* but not with *B. miyamotoi*.

**Conclusions:**

Given the widespread distribution of *B. miyamotoi*, infection due to this agent should be considered in patients who have been exposed to blacklegged ticks in Canada.

## Background

*Borrelia miyamotoi* was first described in *Ixodes persulcatus* ticks and in the blood of rodents collected in Japan in the early 1990s [1]. Subsequently, *B. miyamotoi* was detected, for the first time in North America, associated with blacklegged ticks, *Ixodes scapularis* in several states in the Northeastern United States [2]. Infection rates in field-collected nymphal *I. scapularis* were 1.9-2.5% [2] and unlike the agent of Lyme disease, *Borrelia burgdorferi*, *B. miyamotoi* is transmitted vertically from infected female *I. scapularis* to a variable proportion of larval progeny [2,3]. Initially the public health significance of *B. miyamotoi* was poorly understood; however, recent studies in Russia demonstrate that Old World strains of *B. miyamotoi*, transmitted by *I. persulcatus* cause an influenza-like illness with relapsing fever [4]. In North America, meningoencephalitis was recently described in an elderly immunocompromised patient [5] and results of a serosurvey of patients from southern New England and New York demonstrate that *B. miyamotoi* infection can cause a viral-like illness [6]. These studies support the contention that *B. miyamotoi* is yet another of the guild of pathogens, which includes the agents of Lyme disease, anaplasmosis, babesiosis, Powassan virus and the *Ehrlichia muris*-like agent, associated with blacklegged ticks in North America [7]. The discovery of DNA of *B. miyamotoi* in ticks during a study of *B. burgdorferi* diversity signaled the possible occurrence of *B. miyamotoi* in Canada [8].

Passive surveillance for blacklegged ticks, which involves the submission of ticks collected by the general public and participating medical and veterinary clinics, has been conducted across Canada (excluding British Columbia) since the 1990s [9,10]. Ticks identified as *I. scapularis* have been routinely tested for infection with *B. burgdorferi* and *Anaplasma phagocytophilum* using first a multiplex real-time PCR assay [11], followed by an *ospA* real-time PCR to confirm *B. burgdorferi* infection [9]. Most *I. scapularis* are submitted from locations where reproducing populations of *I. scapularis* occur (southern Manitoba, southern and eastern Ontario, southern Quebec, and locations in New Brunswick and Nova Scotia). Some *I. scapularis* are also submitted from locations where populations are not known to occur (e.g. Alberta, Saskatchewan, Prince Edward Island and Newfoundland) and it is thought that these ticks are ‘adventitious’ ticks dispersed from tick populations in Canada and the USA by migratory birds [9]. Each year a small number of *I. scapularis* submitted in passive surveillance test positive for *Borrelia* species infection in the 23S rRNA real-time PCR screening assay but are negative for *B. burgdorferi* infection in the confirmatory o*spA* real-time PCR assay. Subsequent testing by nested PCR (nPCR) and sequencing indicate that some of these extracts are positive for infection with *B. miyamotoi*. This supports earlier Multilocus Sequence Typing (MLST) analysis in which a small number of *B. burgdorferi*-infected ticks were found to be co-infected with *B. miyamotoi*[8].

We have developed and evaluated molecular assays to identify *B. miyamotoi* and a PCR-based testing protocol or diagnostic approach for testing *I. scapularis* ticks collected in surveillance for tick-borne agents. Data on the western blacklegged tick *I. pacificus*, and *B. miyamotoi* prevalence were not addressed by this study. Here we undertake a systematic analysis of ticks recently collected in surveillance in Canada to i) better understand the possible geographic range of *B. miyamotoi* in Canada; ii) estimate the prevalence of *B. miyamotoi* infection in *I. scapularis* ticks in Canada; and iii) investigate the frequency of co-infections with *B. miyamotoi*, *B. burgdorferi* and *A. phagocytophilum* in *I. scapularis* in Canada.

## Methods

### Development of *B. miyamotoi*-specific IGS real-time PCR

DNA from 25 ticks collected in surveillance prior to 2012 that tested positive with the screening 23S rRNA real-time PCR, but negative with the confirmatory *ospA* real-time PCR was tested with a nPCR specific to the genus *Borrelia*[12] which amplifies 587 bp of the 16S-23S IGS region. For this PCR, 5 μl DNA template was added to 95 μl master mix containing 0.2 mM each dNTP, 0.5 μM forward and reverse primers, 5 Units of AmpliTaq Gold® polymerase and 1.5 mM MgCl_2_ (Life Technologies, Carlsbad, CA). The thermocycler conditions used were as follows: denaturation at 94°C for 4 minutes, 35 cycles of amplification at 94°C for 1 minute, 50°C for 1 minute and 72°C for 1 minute, followed by a 10 minute extension phase at 72°C for both stages of the nested PCR reaction. Amplification products were analyzed by ethidium bromide-stained 2% agarose gels. All nPCR products were purified using Montage®PCR filter units (Millipore) and sequenced on an ABI 3130xl Genetic Analyzer using BigDye™ Terminator version 3.1 cycle sequencing kits. Sequence data was analyzed using DNASTAR Lasergene 9 Software and multiple alignments were performed using Clustal W. Sequences were compared to those in GenBank and BLAST results indicated that 8 of the 25 tick extracts were positive for *B. miyamotoi.* Subsequently, *B. miyamotoi*-specific and *B. burgdorferi*-specific primers and FAM-labeled probes annealing to the 16S-23S IGS were designed from these sequences for real-time IGS PCR (Table 1).

**Table 1:** **Primer and probe sequences for the detection *of A. phagocytophilum *and *Borrelia *species**

		**Primer/Probe**	**5′-3′ nucleotide sequence**
**Duplex screening assay**			
		Bb23Sf	CGAGTCTTAAAAGGGCGATTTAGT
*B. burgdorferi*	23S rRNA	Bb23Sr	GCTTCAGCCTGGCCATAAATAG
		Bb23S-P	FAM-AGATGTGGTAGACCCGAAGCCGAGTG-TAMRA
		ApMSP2f	ATGGAAGGTAGTGTTGGTTATGGTATT
*A. phagocytophilum*	*msp2*	ApMSP2r	TTGGTCTTGAAGCGCTCGTA
		ApMSP2-P	VIC-TGGTGCCAGGGTTGAGCTTGAGATTG-TAMRA
*B. burgdorferi *confirmatory assay(s)*			
		ospAF	CTGGGGAAGTTTCAGTTGAAC
*B. burgdorferi*	*ospA*	ospAR	TTGGTGCCATTTGAGTCGTA
		ospA-P	FAM-CTGCAGCTTGGAATTCAGGCACTT-BBQ
		BbIGSf	AAGAAGGACAAGTATTGTAGCGAG
*B. burgdorferi*	IGS*	BbIGSr	GCAATCTTTGCCTTCCTCC
		BbIGS-P	FAM-TGCCAGTATTTAGTGGTAGGGATTCGG-BBQ
***B. miyamotoi *** **assays**			
		BmiyaIGSf	CGTCTTGTTGCTTTTAAAGTGT
*B. miyamotoi*	IGS	BmiyaIGSr	CATGATCAGGTCCTTGATAATATG
		BmiyaIGS-P	FAM-TGGATTCCAAATTTGATTACATGCAA-BBQ
		MGlpQF	GATAATATTCCTGTTATAATGC
*B. miyamotoi*	*glpq*	MGlpQR	CACTGAGATTTAGTGATTTAAGTTC
		MYS-P	FAM-CCCAGAAATTGACAACCACAAATGT-BHQ2
***Borrelia *** **spp. Nested PCR**			
16S-23S IGS	1st stage	rrs	GTATGTTTAGTGAGGGGGGTG
		rrl	GGATCATAGCTCAGGTGGTTAG
	2nd stage	Fn	AGGGGGGTGAAGTCGTAACAAG
		Rn	GTCTGATAAACCTGAGGTCGGA
Flagellin	1st stage	FO1	AAGTAGAAAAAGTCTTAGTAAGAATGAAGGA
		FO2	AATTGCATACTCAGTACTATTCTTTATAGAT
	2nd stage	FI1	CACATATTCAGATGCAGACAGAGGTTCTA
		FI2	GAAGGTGCTGTAGCAGGTGCTGGCTGT

*B. burgdorferi IGS real-time PCR is performed solely for validation of the specificity of *B. miyamotoi* real-time PCR assays and is not routinely used in the testing protocol.

### Validation of real-time PCR

Validation of the species-specific IGS real-time PCR assays was performed using DNA from the eight 23S PCR-positive and o*spA* PCR-negative ticks mentioned previously, DNA from 72 ticks (collected from 2008 to 2012) that were positive on both the 23S and the *ospA* real-time PCR confirming *B. burgdorferi* infection, and DNA from 9 ticks that were 23S PCR-positive and o*spA* PCR-negative and had been confirmed as being infected with *B. bissettii* by flagellin nPCR. DNA from cultures of *B. garinii* strain ATCC® 51991™ and *B. afzelii* strain ATCC® 51567™, and *B. hermsii* DNA from a clinical sample was used to evaluate the specificity of the assays. Once validated, the species-specific real-time PCR assays were applied to DNA from an additional 39 23S PCR-positive and o*spA* PCR-negative ticks collected during 2008–2012.

Reaction mixtures were prepared in 2x TaqMan**®** Universal Mastermix (Applied Biosystems, Life Technologies) to contain 300 to 600 nM of each primer and 200 nM probe. Amplification was carried out on either an ABI 7500 Real-time PCR System, ABI 7900HT or ABI ViiA7 using 96 well optical plates. Thermocycling conditions consisted of: activation of AmpErase at 50°C for 2 minutes, 10 minutes at 95°C for denaturation of AmpErase and activation of AmpliTaq Gold® Polymerase, followed by 40 cycles of amplification with denaturation at 95°C for 15 seconds and annealing at 58°C for 1 minute. Following amplification and real-time data acquisition, analysis was performed using the Sequence Detection System software. A second real-time PCR assay targeting *B. miyamotoi glpQ*[13] was performed as a confirmatory assay, using an annealing temperature of 50°C.

DNA from *B. miyamotoi-*positive, *B. bissettii*-positive and most *B. burgdorferi*-positive ticks was tested using the nested 16S-23S IGS PCR to generate products for sequencing. This sequence data was considered the gold standard and provided validation data for the *B. miyamotoi* real-time PCR assays.

### Analysis of infections and co-infections in *I. scapularis* ticks collected in passive surveillance

**Figure 1:**
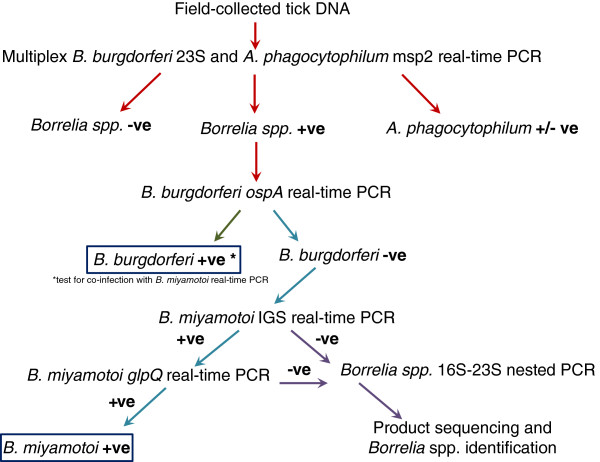
**Testing protocol to detect *B. burgdorferi*, *A. phagocytophilum *** **and *B. miyamotoi *** **in *I. scapularis *** **ticks.**

A total of 4938 *I. scapularis* ticks collected in passive surveillance in 2012 (excluding the 68 used in validation) were tested using the developed testing protocol (Figure 1). The screening 23S and confirmatory o*spA* real-time PCR were used to assess *B. burgdorferi* infection as described above. The screening 23S PCR is a multiplex assay that also detects the presence of *A. phagocytophilum* DNA using primers specific for the *msp2* gene (ApMSP2f and ApMSP2r) [14]. Confirmation of infection with *A. phagocytophilum* is achieved by an in-house real-time PCR assay targeting 16S rRNA. The *Borrelia miyamotoi-*specific IGS real-time PCR was used to detect *B. miyamotoi* infections that were subsequently confirmed by *B. miyamotoi glpQ* real-time PCR. All real-time PCR assays were conducted using the conditions described above for *B. miyamotoi* IGS. Tick extracts that were positive for *Borrelia* spp. infection in the screening 23S real-time PCR, but negative for *B. burgdorferi* in the o*spA* real-time PCR, and negative in the *B. miyamotoi* IGS assay, were tested by 16S-23S IGS nPCR with the aim of sequencing products to identify other infecting *Borrelia* species.

Associations of infections and co-infections in ticks with province of origin, level of engorgement of the tick, host of origin, and tick instar were investigated by logistic regression in STATA version 11.0 for Windows (STATACorp, College Station, TX, USA). The most parsimonious multivariable model was created by backwards and forwards elimination and substitution of variables. Logistic regression models were used to investigate whether or not there were significant associations between infections of ticks with different pathogens. The level of significance throughout was P < 0.05.

## Results

### Development and validation of *B. miyamotoi*-specific IGS real-time PCR

Primers were developed using a sequence with 100% similarity to a sequence from *B. miyamotoi* (GenBank:AY531879) obtained in the 16S-23S IGS nPCR. All 8 (100%) of the *B. miyamotoi*-positive ticks, that were so determined by 16S-23S IGS nPCR and sequencing, were positive by *B. miyamotoi* IGS and *glpQ* real-time PCR. These extracts were also negative in the *B. burgdorferi* IGS real-time PCR assay. All 72 (100%) of the *B. burgdorferi* extracts were positive in the *B. burgdorferi* IGS real-time PCR. Two of 72 (2.8%) of these extracts were also reactive with *B. miyamotoi* IGS and *glpQ* real-time PCR indicating co-infection. The nine extracts known to be positive for *B. bissettii*, DNA from cultures of *B. afzelii* and *B. garinii* did not react in either the *B. burgdorferi* or *B. miyamotoi* IGS or *glpQ* real-time PCR assays. DNA of *B. hermsii* did not react in either the *B. miyamotoi* IGS or *glpQ* real-time PCR but produced a late amplification product (Ct >39) in the *B. burgdorferi* IGS real-time PCR. Of the 39 23S rRNA-positive o*spA*-negative ticks obtained in 2008–2012, 31 (79.5%) were positive for *B. miyamotoi*, while one extract was also co-infected with *B. bissettii*. In total, 7/39 (17.9%) of the extracts were negative by IGS real-time PCR and were subsequently identified as *B. bissettii* by sequencing of products of the 16S-23S IGS nPCR. One of the 39 extracts was positive for *B. burgdorferi* by IGS real-time PCR and subsequently confirmed with 16S-23S IGS sequencing. The reactivity profiles of *B. burgdorferi*, *B. miymotoi* and *B. bissettii* in the various PCR assays (Table 2) serve as the basis for our testing protocol to detect the suite of *Borrelia* species found in blacklegged ticks collected in Canada.

**Table 2:** **PCR reactivity profiles for *Borrelia *species detected in *I. scapularis *ticks in Canada**

	***Borrelia *species**		
**PCR reaction**	***B. burgdorferi***	***B. miyamotoi***	***B. bissettii***
Real-time PCR			
23S rRNA	*+*	*+*	*+*
*ospA*	*+*	*-*	*-*
*B.burgdorferi* IGS	*+*	*-*	*-*
*B. miyamotoi* IGS	*-*	*+*	*-*
*B. miyamotoi glpQ*	*-*	*+*	*-*
Conventional nested PCR			
Flagellin	*+*	*-*	*+/−*
*ospA*	*+*	*-*	*+/−*
16S-23S IGS	*+*	*+*	*+*

### Analysis of infections and co-infections in *I. scapularis* ticks collected in passive surveillance

Of the 4938 ticks tested (Table 3), 41 (0.8%) were infected with *A. phagocytophilum* (0/4 larvae, 0/139 nymphs, 37/4778 adults), 696 (14.1%) were infected with *B. burgdorferi* (0/4 larvae, 16/139 nymphs, 676/4778 adults) and 23 (0.5%) were infected with *B. miyamotoi* (0/4 larvae, 1/139 nymphs, 22/4778 adults) (Tables 4 and 5). No other *Borrelia* spp. were detected in the ticks.

**Table 3:** **Collection data by province for *I. scapularis *ticks submitted through passive surveillance in 2012**

**Province**	**Total no. ticks**	Instar*			**State of engorgement **^**†**^		**Host**			
		**Larvae**	**Nymphs**	**Adults**	**Unfed**	**Fed**	**Dog**	**Cat**	**Human**	**Other**
AB	87			87		87	78	9		
MB	170		13	152	51	115	98	16	53	1
ON	2591	4	97	2482	1397	1183	445	28	2102	7
NB	366		14	350	76	285	187	21	123	7
NL	33		1	32	3	30	20	10	3	
NS	34			34	9	24	21		13	
PEI	178		1	176	8	168	139	28	11	
QC	1479		13	1465	297	1179	919	129	427	3
Total	4938	4	139	4778	1841	3071	1907	241	2732	18

*Of the adult ticks 61 were males the rest females and for 17 ticks the instar was not recorded.

^†^For 26 ticks the state of engorgement was not recorded.

AB = Alberta, MB = Manitoba, ON = Ontario, NB = New Brunswick, NL = Newfoundland & Labrador, NS = Nova Scotia, PEI = Prince Edward Island, QC = Quebec, Ap = A*. phagocytophilum*, Bb = *B. burgdorferi* and Bm = *B. miyamotoi.*

**Table 4:** Prevalence of infection of ticks* with *A. phagocytophilum*, *B. burgdorferi *and *B. miyamotoi *by province

**Province total no. ticks**		**Number (%) infected**		**Number (%) co-infected**			
		**Ap**	**Bb**	**Bm**	**Ap-Bb**	**Ap-Bm**	**Bb-Bm**
AB	87	5 (5.7)	12 (13.8)	1 (1.1)	1 (1.1)		
MB	170	5 (2.9)	15 (8.8)	2 (1.2)	3 (1.8)		
ON	2591	7 (0.3)	411 (15.9)	7 (0.3)	2 (0.08)		2 (0.08)
NB	366	3 (0.8)	25 (6.8)	3 (0.8)	1 (0.3)		
NL	33	1 (3.0)	9 (27.3)		1 (3.0)		
NS	34		4 (11.8)	1 (2.9)			1 (2.9)
PEI	178	1 (0.6)	17 (9.6)	1 (0.6)			
QC	1479	19 (1.3)	203 (13.7)	8 (0.5)	3 (0.2)		5 (0.3)
Total	4938	41 (0.8)	696 (14.1)	23 (0.5)	11 (0.2)	0	8 (0.2)

*I. scapularis ticks collected by passive surveillance in 2012.

**Table 5:** **Prevalence of infection of *I. scapularis *ticks**^**† **^by instar*

	**Tick Instar**		
**Pathogen**	**Larvae**	**Nymphs**	**Adults**
*B. miyamotoi*	0/4 (0)	1/139 (0.7)	22/4778 (0.5)
*B. burgdorferi*	0/4 (0)	16/139 (11.5)	676/4778 (14.1)
*A. phagocytophilum*	0/4 (0)	0/139 (0)	37/4778 (0.8)
Coinfections of *B. miyamotoi* and *B. burgdorferi*	0/4 (0)	0/139 (0)	8/4778 (0.2)
Coinfections of *A. phagocytophilum* and *B. burgdorferi*	0/4 (0)	0/139 (0)	11/4778 (0.2)

*This table does not include data from 17 ticks for which instar was not recorded.

^†^Collected by passive surveillance in 2012.

*Borrelia miyamotoi* was found in blacklegged ticks from all provinces except Newfoundland, and there were no significant variations amongst provinces in the prevalence of *B. miyamotoi* infection of ticks. All other explanatory variables were not significantly associated with *B. miyamotoi* infection.

There were significant variations amongst provinces in the likelihood that a tick was positive for *B. burgdorferi*. The provinces could be simplified into three groups within which the prevalence of infection was similar: i) Alberta and Manitoba, ii) New Brunswick and Prince Edward Island, and iii) ticks from all other provinces (Ontario, Quebec, Nova Scotia and Newfoundland) (χ^2^ = 4.8, df = 5, P > 0.1). Ticks from New Brunswick and Prince Edward Island were significantly less likely to be infected with *B. burgdorferi* than ticks from Ontario, Quebec, Nova Scotia and Newfoundland combined (OR = 0.55, 95% CI = 0.39 – 0.78, P < 0.01), and less likely to be infected than ticks in Alberta and Manitoba (by Wald test of final model parameters: χ ^2^ = 14.7, df = 1, P < 0.001). Ticks from Alberta and Manitoba were less likely to be infected with *B. burgdorferi* than ticks from Ontario, Quebec, Nova Scotia and Newfoundland combined (OR = 0.64, 95% CI = 0.41 – 0.98, P < 0.05). Ticks were significantly less likely to be infected if they fed on humans (OR = 0.59, 95% CI = 0.46 – 0.75, P < 0.001), and were less likely to be infected if they were slightly, partially or fully engorged than if they were unfed (ORs = 0.44, 0.30, 0.21; 95% CIs = 0.33-0.60, 0.24-0.39, 0.10-0.44; P < 0.001 for all).

No immature ticks were infected with *A. phagocytophilum* but there was significant variation amongst province of origin in the proportion of adult ticks infected with *A. phagocytophilum*. Adult ticks collected in Alberta and Manitoba were significantly more likely to be infected than ticks from other locations (OR = 4.5, 95% CI = 2.0-10.4, P < 0.001). Ticks from Quebec were significantly more likely to be infected than ticks from Ontario, New Brunswick, Nova Scotia and Newfoundland combined (OR = 3.7, 95% CI = 1.7-7.7, P < 0.001). There were no significant variations in prevalence of *A. phagocytophilum* infection associated with state of engorgement or host of origin.

Co-infections were detected in 19 ticks (15 being adult females, one being an adult male and 3 having the instar unrecorded), of which 11 (0.23% of adult ticks) were co-infected with *A. phagocytophilum* and *B. burgdorferi*, and 8 (0.17% of adult ticks) were co-infected with *B. burgdorferi* and *B. miyamotoi* (Tables 4 and 5). Consequently statistical analysis was limited to adult ticks. Adult ticks were significantly more likely to be infected with *B. miyamotoi* if they were infected with *B. burgdorferi* (OR = 3.5, 95% CI = 1.5 – 8.4, P < 0.01). This relationship remained significant when other variables (province of origin, level of engorgement of the tick, host of origin, and tick instar) were included in the model. There was no significant association between *A. phagocytophilum* and *B. burgdorferi* infection in adult ticks.

## Discussion

The objective of this study was to develop and implement a systematic approach using real-time PCR assay to detect *B. miyamotoi*, *B. burgdorferi* and *A. phagocytophilum* infections and co-infections in ticks collected in surveillance. In doing so, we were able to assess the prevalence of infection of ticks collected in Canada, with the newly-recognized pathogen *B. miyamotoi*. Results of our study identified *B. miyamotoi*-infected ticks at low (<1%) prevalence in most provinces. Few ticks were co-infected, however a third of *B. miyamotoi*-infected ticks and a quarter of *A. phagocytophilum-*infected ticks were also infected with *B. burgdorferi* and co-infections of *B. miyamotoi* and *B. burgdorferi* occurred more frequently than would be expected by chance.

It is increasingly recognized that *I. scapularis* ticks transmit a range of bacteria including the Lyme disease-causing *B. burgdorferi*, *B. miyamotoi*, the *E. muris*-like agent [15], and *A. phagocytophilum*[16] as well as bacteria such as *B. carolinensis*[17] and *B. bissettii*[18] whose pathogenicity has not yet been determined. Here we have developed new assays and combined them with existing ones to create a PCR testing protocol, similar to that of Ullman *et al*. [13], which allowed us to detect and identify infections and co-infections of ticks with different *Borrelia* species and *A. phagocytophilum*. The new 16S-23S IGS real-time PCR assays were robust showing 100% concordance between positive results and sequence analysis indicating high specificity. There was slight reactivity of *B. hermsii* DNA in the *B. burgdorferi* IGS real-time assay, but this is of little consequence on test outcomes as *B. hermsii* is transmitted by Argasid ticks and would rarely, if ever, be encountered in blacklegged ticks obtained in surveillance. Future refinements of this testing protocol will include implementation of a duplex real-time PCR assay for *B. burgdorferi ospA* and *B. miyamotoi* 16S-23S IGS to reduce PCR steps, development of a real-time PCR assay to detect *B. bissettii* and incorporation of PCR assays for non-bacterial *I. scapularis*-borne pathogens such as Powassan encephalitis virus.

By implementation of this new testing protocol for the detection of selected species of *Borrelia*, we have expanded on the findings of Ogden *et al*. [8] who first detected *B. miyamotoi* in blacklegged ticks collected in Canada. The prevalence of *B. miyamotoi* in blacklegged ticks in our study (<1%) was lower than the 1 – 5% reported in the eastern USA [2,3], and this difference may suggest that *B. miyamotoi* transmission cycles are at an early stage of becoming established amongst resident tick and rodent populations in Canada compared to localities in the USA [19]. Infection prevalence of tick-borne pathogens in ticks and hosts may take some years to rise to an equilibrium level, particularly if the ticks are at low densities, being themselves at an early stage of becoming established [20]. However, our study also confirms that as in the US, *B. miyamotoi* can be detected across the geographic range of *I. scapularis* in Canada [2,3,8]. Geographic variations in the prevalence of *B. burgdorferi* and *A. phagocytophilum* infection in ticks were detected and these are consistent with previous studies. Low *B. burgdorferi* infection prevalence was detected in ticks from New Brunswick and Manitoba, which is consistent with previous analyses linking low *B. burgdorferi* infection prevalence in *I. scapularis* populations that are emerging in these locations [21,22], as well as in provinces where ticks from these new populations are likely carried by migratory birds (from New Brunswick into Prince Edward Island where no deer occur to permit ticks to establish, and from Manitoba into Alberta). There were no ticks submitted from Saskatchewan during this study period and ticks from Alberta would be expected to be ‘adventitious’ ticks dispersed by migratory birds from Manitoba or the upper Mid-West of the USA [8,9,22]. Higher infection prevalence of *A. phagocytophilum* in ticks from Manitoba and Alberta is consistent with spring synchrony of larval and nymphal *I. scapularis* tick activity in the west of the tick’s range enhancing transmission of short-lived rodent host infections compared to the more asynchronous transmission in the east [20,23]. Higher infection *A. phagocytophilum* prevalence in ticks in some locations in Quebec has been detected possibly associated with founder events in naïve host populations [24,25]. In contrast to *B. burgdorferi*, there was no evidence of geographic variation (in the presented analysis as well as in cluster analysis not described here) in the prevalence of *B. miyamotoi* infection of ticks, which is consistent with more simultaneous introduction of *B. miyamotoi* with *I. scapularis*. As *B. miyamotoi* is transovarially and transtadially transmitted in ticks, this bacterium can be imported in host-dispersed infected engorged nymphal ticks as well as larval ticks, while only imported engorged larvae can efficiently introduce *B. burgdorferi*[22].

Variations in prevalence of *B. burgdorferi* infection with stage of engorgement are consistent with our findings in previous studies; *B. burgdorferi* multiplies in the tick as it feeds [21]. Variations in prevalence of *B. burgdorferi* infection with host of origin have also been observed in our passive surveillance data [21] although here ticks collected from humans were less likely to be detected as infected. The underlying reason for this variation is unknown; however an analysis of the quality of the submitted ticks did not indicate significant differences between ticks removed from humans or companion animals (data not shown). It is possible that ticks from dogs had higher infection prevalence than ticks from humans because some of the dogs were infected and consequently infective for ticks that fed on them [26]. Some *I. scapularis* ticks were co-infected with *B. miyamotoi* and *B. burgdorferi* or with *A. phagocytophilum* and *B. burgdorferi* however, co-infection with *B. miyamotoi* and *B. burgdorferi* occurred more frequently than by chance, which is consistent with shared reservoirs for these species [20,27]. The implications of these observations for disease in humans are at present unknown and require further investigation, as does the occurrence of *B. miyamotoi* in *I. pacificus* ticks, the other main vector of tick-borne zoonoses that occurs in British Columbia.

## Conclusions

The relatively limited (though expanding) distribution of blacklegged tick populations in Canada [22,28,29] and the lower prevalence of *B. miyamotoi* infection in these ticks means that at present the risk of infection of humans in Canada would be lower than in parts of the USA [6]. Nevertheless, our study indicated that *B. miyamotoi* is present across a wide geographic range in Canada, and clinicians should consider *B. miyamotoi* infection as a possible diagnosis, alongside Lyme disease, Anaplasmosis, Ehrlichiosis, Babesiosis and arboviral infections, in patients suffering from suspected infectious disease who have potentially been exposed to ticks in Canada. Our findings underline the need for improved diagnostics for *B. miyamotoi* and other tick-borne pathogens, and ongoing exploration for novel tick-borne pathogens.

## Competing interests

The authors declare that they have no competing interests.

## Authors’ contributions

All authors have contributed significantly to either the bench work, the design of the study, data analysis or drafting of the manuscript. All authors read and approved the final version of the manuscript.
